# Editorial: Special Issue “Satellite Networks for Massive IoT Communication”

**DOI:** 10.3390/s22114214

**Published:** 2022-06-01

**Authors:** Riccardo De Gaudenzi, Beatriz Soret

**Affiliations:** 1 European Space Agency, European Space and Technology Center (ESTEC), Keplerlaan 1, P.O. Box 299, 2200 AG Noordwijk, The Netherlands; 2 Department of Electronic Systems, Aalborg University, Fredrik Bajers Vej 7, C1-1119220 Aalborg, Denmark; bsa@es.aau.dk

The growing need to interconnect sensors and devices anytime, anywhere is stimulating the design and development of innovative solutions for efficiently supporting a wide variety of applications: from simple low-duty cycle sensors for which energy efficiency and low-cost are of paramount importance, to more demanding platforms (e.g., automotive) hosting several sensors requiring telemetry support with global coverage. Among other challenges, these Internet of Things (IoT) or massive Machine Type Communication (mMTC) scenarios entail providing access to a massive number of devices. Terrestrial networks are increasingly expanding their capability to support this kind of IoT services through 4G/5G cellular networks operating in licensed bands and low power wide area networks (LPWAN) operating in unlicensed bands. These deployments will be complemented with satellite networks in order to cover areas where terrestrial service cannot be provided and ensure full ubiquity. This requires dedicated research and solutions for the limits imposed by satellites when providing massive access and the increased coverage area. In recent years, we witnessed a blossoming in research about random access techniques for satellite communications. Interestingly, some of the new ideas developed found practical applications in satellite networks and have been triggering attention in the terrestrial wireless community for massive access. This Special Issue of *Sensors* is aiming at collecting state-of-the-art research papers related to random access techniques and technologies for satellite communications.

In this special issue of *Sensors* we have been compiling a selection of contributions addressing the massive satellite IoT provision technical aspects from analytical and implementation perspectives.

In [1] Arcidiacono et al. show that non-orthogonal uncoordinated multiple access is already a reality in satellite IoT networks thanks to the rapid adoption of the enhanced spread ALOHA (E-SSA) random access technique by industry. It is shown that the developed solution, based on an E-SSA random access technique, achieves an unprecedented throughput, scalability, and service cost, and is well matched to several mMTC satellite use cases.

Munari and Clazzer in [2] are investigating from a theoretical perspective the coexistence of a quality of service (QoS)-constrained service with IoT traffic in a shared spectrum as alternative to a more traditional orthogonal allocation among the two services, looking at possible technique application to satellite applications. The potential benefit of an overlay allocation with respect to segregating the resources for the two services is analytically shown.

In [3], Caus et al. propose a grant-free access scheme called resource sharing beamforming access (RSBA) and investigates its application for low Earth orbit (LEO) satellite communications exploiting massive multiple-input multiple-output (MIMO). Through analysis, it is shown that RBSA reduces the probability of collision, and thus it increases the number of terminals that can access the media.

In [4], Isca et al. provide an overview of recent results of a study looking at the design, development and performance evaluation study of satellite gateways to receive and manage the traffic from a large population of uncoordinated IoT user terminal. In this case, the RA aggregate throughput is further enhanced by a minimum mean-square error (MMSE) linear detector stage in front of the E-SSA demodulator recursive successive interference cancellation.

Temim, Ferré, and Tjan in [5] propose an enhancement of the popular terrestrial LoRa LPWAN ultra-narrow-band physical layer to better cope with satellite Doppler shift occurring in LEO IoT constellations. Numerical results confirm the suitability of the proposed differential chirp spread spectrum modulation for LEO IoT applications.

Finally, we would like to thank all the authors for their excellent contributions and the reviewers for their fruitful comments and feedback. Furthermore, we wish to express my appreciation to the excellent support by the Editorial Board and Editorial Office of the MDPI *Sensors* journal.

## Data Availability

Not applicable.
